# A Surface Groove Essential for Viral Bcl-2 Function During Chronic Infection In Vivo

**DOI:** 10.1371/journal.ppat.0010010

**Published:** 2005-09-30

**Authors:** Joy Loh, Qiulong Huang, Andrew M Petros, David Nettesheim, Linda F. van Dyk, Lucia Labrada, Samuel H Speck, Beth Levine, Edward T Olejniczak, Herbert W. Virgin

**Affiliations:** 1 Departments of Pathology and Immunology and Molecular Microbiology, Washington University School of Medicine, St. Louis, Missouri, United States of America; 2 Pharmaceutical Discovery Division, Abbott Laboratories, Abbott Park, Illinois, United States of America; 3 Department of Microbiology, University of Colorado Health Science Center, Aurora, Colorado, United States of America; 4 Department of Medicine, Columbia College of Physicians and Surgeons, New York, New York, United States of America; 5 Division of Microbiology and Immunology, Yerkes Regional Primate Center, Emory University, Atlanta, Georgia, United States of America; 6 Departments of Internal Medicine and Microbiology, University of Texas Southwestern Medical Center, Dallas, Texas, United States of America; University of Western Ontario, Canada

## Abstract

Antiapoptotic Bcl-2 family proteins inhibit apoptosis in cultured cells by binding BH3 domains of proapoptotic Bcl-2 family members via a hydrophobic BH3 binding groove on the protein surface. We investigated the physiological importance of the BH3 binding groove of an antiapoptotic Bcl-2 protein in mammals in vivo by analyzing a viral Bcl-2 family protein. We show that the γ-herpesvirus 68 (γHV68) Bcl-2 family protein (γHV68 v-Bcl-2), which is known to inhibit apoptosis in cultured cells, inhibits both apoptosis in primary lymphocytes and Bax toxicity in yeast. Nuclear magnetic resonance determination of the γHV68 v-Bcl-2 structure revealed a BH3 binding groove that binds BH3 domain peptides from proapoptotic Bcl-2 family members Bax and Bak via a molecular mechanism shared with host Bcl-2 family proteins, involving a conserved arginine in the BH3 peptide binding groove. Mutations of this conserved arginine and two adjacent amino acids to alanine (SGR to AAA) within the BH3 binding groove resulted in a properly folded protein that lacked the capacity of the wild-type γHV68 v-Bcl-2 to bind Bax BH3 peptide and to block Bax toxicity in yeast. We tested the physiological importance of this v-Bcl-2 domain during viral infection by engineering viral mutants encoding a v-Bcl-2 containing the SGR to AAA mutation. This mutation resulted in a virus defective for both efficient reactivation of γHV68 from latency and efficient persistent γHV68 replication. These studies demonstrate an essential functional role for amino acids in the BH3 peptide binding groove of a viral Bcl-2 family member during chronic infection.

## Synopsis

Viruses can manipulate their hosts by expressing proteins that structurally and functionally resemble host cellular proteins. One important cellular process manipulated by viruses is apoptosis, a cell death program that is regulated by a family of Bcl-2-like proapoptotic and antiapoptotic proteins. Gammaherpesviruses encode Bcl-2 family proteins (v-Bcl-2) that may contribute to their ability to cause tumors and persist for the lifetime of their hosts. The authors solved the structure of the murine γ-herpesvirus 68 (γHV68) v-Bcl-2 and found that it is similar to cellular antiapoptotic proteins and that v-Bcl-2 uses the same mechanism as cellular Bcl-2 to bind to peptides from proapoptotic Bcl-2 family proteins. Furthermore, they found that a γHV68 virus expressing a mutated form of v-Bcl-2 that cannot bind to peptides from proapoptotic Bcl-2 family proteins is defective in its ability to cause chronic viral infection in mice. Thus, a specific structural feature and molecular mechanism of the v-Bcl-2 that is shared with host antiapoptotic Bcl-2 proteins is important for the function of this protein during viral infection. These findings enhance our understanding of the molecular mechanisms of chronic γ-herpesvirus infection, and suggest that targeting the functions of the v-Bcl-2 protein might have therapeutic benefit.

## Introduction

Bcl-2 family proteins are important regulators of cell death and other aspects of cell physiology such as glycolysis and calcium metabolism [1–5]. The Bcl-2 family can be divided into antiapoptotic proteins, which have in common four Bcl-2 homology domains (BH1–4), and proapoptotic proteins, which have either BH1–3 domains or only a BH3 domain [2–4,6–8]. A key mechanism by which this family regulates cell death in cultured cells involves a binding interaction between a hydrophobic groove on the surface of the antiapoptotic family protein and the BH3 domain of proapoptotic family members [2–4,9–13]. Furthermore, the importance of BH3 binding domains of antiapoptotic proteins has been shown in vivo in nematodes [14,15]. However, antiapoptotic Bcl-2 family proteins also interact with proteins outside of the Bcl-2 family, such as Aven, Apaf-1, Btf, Beclin1, Raf-1, calcineurin, tissue transglutaminase, FAST, and p53 [6,16–26]. The structural basis for interactions between these proteins and Bcl-2 family members, and the functional significance of these interactions, is less well understood than the structural basis and functional importance of interactions among Bcl-2 family members.

Certain viruses have acquired during evolution host genes that confer selective advantages. Viral proteins encoded by these genes often retain or even enhance advantageous functions of their host counterparts and lose functions that do not benefit the virus. Importantly, many viruses encode antiapoptotic Bcl-2 family proteins [7,27,28]. For example, viral Bcl-2 family proteins are encoded by all γ-herpesviruses, including the human viruses Epstein-Barr virus (EBV) and Kaposi's sarcoma-associated herpesvirus (KSHV), and the murine virus γ-herpesvirus 68 (γHV68) [7,27].

The conservation of v-Bcl-2 genes across the γ-herpesviruses suggests that these proteins play an important and evolutionarily conserved role in the pathogenesis of γ-herpesvirus infection. EBV, KSHV, and γHV68 all latently infect B cells [29–31], reactivate from latency, persistently replicate in both normal and immunocompromised hosts, and induce B cell lymphomas during chronic infection ([32] and unpublished data). The capacity to establish and reactivate from viral latency is essential for maintaining the lifelong infection that is characteristic of γ-herpesviruses. In addition, the capacity to persistently replicate at a low level despite the presence of active host immunity is a critically important aspect of γ-herpesvirus pathogenesis. Persistent replication contributes to viral spread from chronically infected hosts to new hosts, and may well contribute to tumorigenesis [33–35].

An in vivo role during infection has been demonstrated only for the γHV68 v-Bcl-2 [36]. The γHV68 v-Bcl-2 protein has been shown to inhibit apoptosis induced by Fas, TNFα, and Sindbis virus infection [37–39]. It is expressed during latency and has critical roles in reactivation from latency, the capacity to persistently replicate in immunocompromised mice such as those lacking interferon-γ (IFNγ), and in determining the number of latent cells early after infection [36,40,41]. Surprisingly, the γHV68 v-Bcl-2 has no role during acute infection of fibroblast cells in vitro or in vivo [36]. These data suggest that viral regulation of apoptosis is more important during chronic than acute γHV68 infection. Further support for this concept comes from studies showing that proapoptotic host molecules such as perforin, granzymes, and caspase 3 are unimportant during acute γHV68 infection but are critically important for limiting the amount of latent γHV68 infection [42,43]. Together, these data suggest that regulation of apoptosis by host and viral genes plays a critical role specific to chronic γHV68 infection.

These observations demonstrate a physiological role for a v-Bcl-2 family member in reactivation from viral latency and in persistent replication. The molecular mechanisms responsible for this role in vivo can be defined by evaluating the phenotypes of viruses expressing mutant forms of the v-Bcl-2 protein. We reasoned that analysis of a viral Bcl-2 protein using a combination of structural, biochemical, and pathogenesis studies would reveal whether viral and host Bcl-2 family members might function via the same mechanisms, and would clarify the importance of those mechanisms during infection. In this study, we identified the structure and biochemical function of a domain in γHV68 v-Bcl-2 predicted to be important for v-Bcl-2 function, and then tested the role of this domain in vivo*.* The γHV68 v-Bcl-2 shared with host antiapoptotic Bcl-2 family proteins the capacity to block apoptosis in primary lymphocytes induced by antigen receptor signaling, corticosteroids, and γ-irradiation, and to inhibit Bax toxicity in yeast. Solving the three-dimensional structure of the v-Bcl-2 revealed a functional hydrophobic surface groove that binds Bax and Bak BH3 peptides via a mechanism shared with host anti-apoptotic Bcl-2 family members. Mutations within this BH3 binding groove significantly decreased binding affinity for Bax peptide, abrogated inhibition of Bax toxicity in yeast, and ablated v-Bcl-2 function during chronic infection. These studies are the first to identify a specific domain of a viral Bcl-2 family protein that is essential for a physiological activity in vivo.

## Results

### γHV68 v-Bcl-2 Inhibits Apoptosis Induced by Diverse Apoptotic Stimuli in Primary Lymphocytes

**Figure 1:**
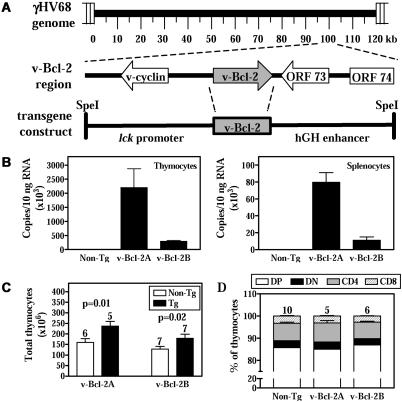
Transgenic Expression of v-Bcl-2 in Thymocytes (A) Schematic illustration of the γHV68 genome, v-Bcl-2 genomic region, and transgene construct. (B) Real-time RT-PCR quantitation of v-Bcl-2 expression in transgenic thymocytes (left) and splenocytes (right). (C) Total number of thymocytes in v-Bcl-2 transgenic and nontransgenic mice. (D) Percentage of CD4 or CD8 single-positive, double-positive (DP) and double-negative (DN) thymocytes in v-Bcl-2 transgenic and nontransgenic mice.

The γHV68 v-Bcl-2 has been shown to block apoptosis in cultured cells in response to several proapoptotic stimuli [37–39]. We evaluated the antiapoptotic activity of the γHV68 v-Bcl-2 in vivo in primary cells by generating transgenic mice expressing v-Bcl-2 in thymocytes under the control of the *lck* proximal promoter (Figure 1A). While this approach can determine the effect of only ectopically expressed v-Bcl-2 in T lymphocytes, it has been successfully used to show the activity of both Bcl-2 and Bcl-x_L_ in primary cells in vivo [44,45]. An added advantage of this system is the ability to study the activity of the v-Bcl-2 using multiple well-characterized lymphotoxic stimuli that have been shown to induce apoptotic cell death [46–51]. No antibodies for the γHV68 v-Bcl-2 are available, so we used quantitative RT-PCR to demonstrate specific γHV68 v-Bcl-2 mRNA expression in two transgenic mouse lines (v-Bcl-2A and v-Bcl-2B). v-Bcl-2A mice had 4- to 6-fold higher v-Bcl-2 expression in thymus and spleen than v-Bcl-2B mice (Figure 1B).

**Figure 2:**
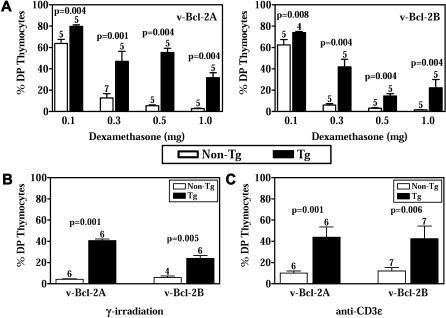
v-Bcl-2 Inhibits Cell Death in Thymocytes (A) Survival of DP thymocytes from v-Bcl-2A (left) and v-Bcl-2B (right) mice 48 h following intraperitoneal injection with various doses of dexamethasone. (B and C) Survival of DP thymocytes from v-Bcl-2A and v-Bcl-2B mice 48 h following (B) 250 rads of γ-irradiation and (C) intraperitoneal injection with 30 μg of anti-CD3ɛ antibody.

γHV68 v-Bcl-2 transgenic mice had higher numbers of total thymocytes than nontransgenic mice (Figure 1C), consistent with an effect of v-Bcl-2 on cell survival. However, v-Bcl-2 transgenic mice displayed no abnormalities in CD4 and CD8 T cell development in the thymus (Figures 1D and S1), nor were there any alterations in thymic architecture (unpublished data). Additionally, v-Bcl-2 transgenic thymocytes did not survive longer in explant culture than control cells (unpublished data). We next determined whether γHV68 v-Bcl-2 inhibits cell death induced by three diverse apoptotic stimuli in vivo. γHV68 v-Bcl-2 transgenic double-positive thymocytes were significantly more resistant than control thymocytes to apoptosis induced by dexamethasone, γ-irradiation, and CD3ɛ ligation (Figures 2 and S1). The increase in survival of transgenic double-positive thymocytes exceeds, and is therefore not attributable to, the slight increase in total thymocyte number in transgenic mice. Transgenic thymocytes from v-Bcl-2A mice were more resistant to dexamethasone than those from v-Bcl-2B mice, particularly at the highest doses of dexamethasone, consistent with higher levels of expression of v-Bcl-2 in these cells (see Figure 1B). No depletion of double-positive thymocytes was observed in transgenic or nontransgenic mice treated with vehicle or isotype antibody controls (unpublished data).

These results are similar to those previously found using host Bcl-2 and Bcl-x_L_ transgenic mice [44,45,52] and show that the γHV68 v-Bcl-2 shares with its cellular counterparts the capacity to inhibit apoptosis induced by diverse proapoptotic stimuli in primary thymocytes. Together with previous studies in cultured cells [37–39], these data strongly support the concept that the γHV68 v-Bcl-2 blocks apoptosis by targeting a step or steps common to the death programs triggered by multiple cellular signals.

### γHV68 v-Bcl-2 Is Structurally Homologous to Bcl-2 and Bcl-x_L_

**Figure 3:**
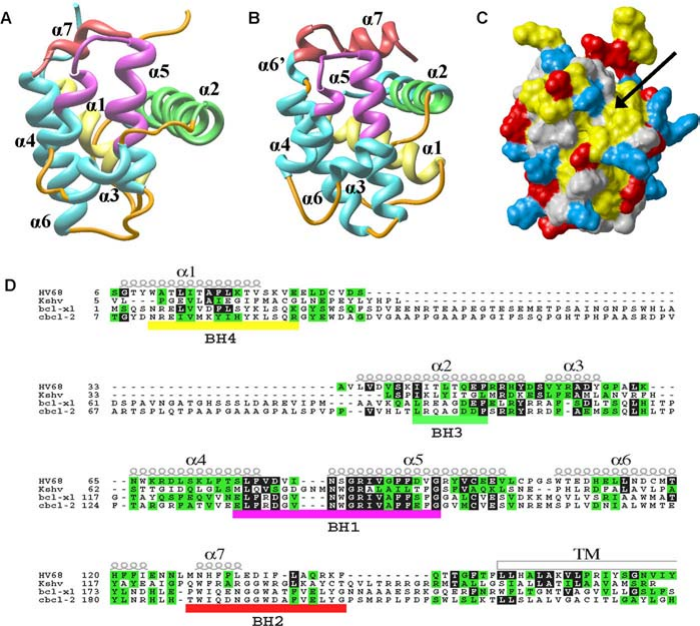
Solution Structure of γHV68 v-Bcl-2 (A and B) Ribbon representation of (A) γHV68 v-Bcl-2 and (B) Bcl-x_L_ with BH1, BH2, BH3, and BH4 regions in magenta, red, green, and yellow, respectively. Helices are numbered with respect to Bcl-x_L_. (C) Connolly surface for γHV68 v-Bcl-2 calculated using a probe radius of 1.4 Å. Residues are colored as follows: Leu, Val, Ile, Phe, Tyr, Trp, Met, and Ala in yellow; Arg, Lys, and His in blue; Asp and Glu in red; all others in gray. The hydrophobic groove is indicated by an arrow. (D) Structural and sequence alignment of KSHV and γHV68 v-Bcl-2 proteins with Bcl-2 and Bcl-x_L_.

The γHV68 v-Bcl-2 has limited amino acid homology to host Bcl-2 family proteins outside of the BH1 domain. Therefore, to understand the molecular basis of v-Bcl-2 function and the relationship between the γHV68 v-Bcl-2 and host Bcl-2 family members, we expressed and purified the protein and determined its structure by nuclear magnetic resonance (NMR) spectroscopy. For structural studies, we expressed amino acids 1–137 of the v-Bcl-2, removing the carboxy-terminal hydrophobic domain. The overall fold of v-Bcl-2 (Figure 3) was very similar to that of Bcl-x_L_ (Figure 3B) [53] and other Bcl-2 family proteins [2]. The core of v-Bcl-2 was formed by two predominantly hydrophobic central helices, α5 and α6, which were flanked on one side by α3 and α4 and on the other side by α1, α2, and α7. The average minimized coordinates for the γHV68 v-Bcl-2 have been deposited with the Protein Data Bank (PDB [http://www.rcsb.org/pdb/] accession code 2ABO).

v-Bcl-2 clearly contained structural elements corresponding to the BH1, BH2, BH3, and BH4 regions of Bcl-x_L_, despite the fact that the v-Bcl-2 BH4 region was not readily apparent in sequence alignments with Bcl-x_L_ (Figure 3D). The BH1 region (in magenta) was composed of the carboxy-terminal end of α4, the loop connecting α4 to α5, and the amino-terminal end of α5. The BH2 region (in red; Figure 3D) was composed of α7, and the BH3 (in green) and BH4 (in yellow) regions were composed of α2 and α1, respectively. The two side chain conformations of Trp10 in α1 generated two distinct sets of resonances for residues in this region, and the resonances corresponding to the predominant conformation were used for these analyses. One notable difference between the γHV68 and KSHV v-Bcl-2 proteins and host antiapoptotic Bcl-2 family proteins is the lack of an extended loop between α1 and α2, which in host Bcl-2 contains sites for caspase cleavage and regulatory phosphorylation [1,2,54].

An alignment revealing the structural and amino acid conservation between Bcl-2, Bcl-x_L_, the γHV68 v-Bcl-2, and the KSHV v-Bcl-2 proteins is shown in Figure 3D. Aside from the lack of the α1/α2 loop in the v-Bcl-2, this structural alignment reveals how a similar overall structure relates to relatively poor amino acid homology in regions outside of the BH1 domain. This striking conservation of structure between v-Bcl-2 and other Bcl-2 family members, despite only low-level amino acid homology outside of the BH1 domain, supports the concept that strong evolutionary pressure has led to retention of the overall structure, and suggests that the mechanisms of v-Bcl-2 action might therefore be similar to those of its cellular counterparts.

### γHV68 v-Bcl-2, Like Antiapoptotic Host Bcl-2 Family Proteins, Has a Hydrophobic Surface Groove and Binds BH3 Peptides from Proapoptotic Bcl-2 Family Members

γHV68 v-Bcl-2 had an elongated surface hydrophobic groove composed of amino acid residues from α2, α3, α4, and α5, similar to the groove observed on Bcl-2 and Bcl-x_L_ (Figures 3C and S2). The orientation of α3 and α4 defined the bottom of this groove, which is the predicted binding site for BH3 domains of proapoptotic Bcl-2 family proteins [2,53]. Conservation of this hydrophobic groove in the γHV68 v-Bcl-2 suggests that it may, like host Bcl-2, bind BH3 domains of proapoptotic Bcl-2 family proteins. One approach to defining the biochemical properties of the surface groove of an antiapoptotic Bcl-2 family member is to measure the affinity of the protein for BH3 peptides from proapoptotic Bcl-2 family members [2–4]. For example, the BH3 domain of Bak is necessary and sufficient for effecting cell death, and mutations in Bak BH3 peptide that reduce binding to Bcl-x_L_ also abrogate interaction of full-length Bak with Bcl-x_L_ [55,56].

We therefore used NMR to analyze binding of BH3 peptides from proapoptotic Bcl-2 family proteins Bax, Bak, and Bad to purified γHV68 v-Bcl-2. We focused on Bak and Bax, since γHV68 v-Bcl-2 blocks apoptosis induced by diverse stimuli (see Figure 2), and Bak and Bax are essential for cell death induced by many stimuli [57–59]. We selected the Bad BH3 peptide for analysis since Bad is a potently proapoptotic BH3-only Bcl-2 family member that induces cell death via interaction with antiapoptotic Bcl-2 family members [8]. Both Bax and Bak peptides were in slow exchange with γHV68 v-Bcl-2 on the NMR timescale, indicating K_d_ values of less than 5 μM. In contrast, Bad BH3 peptide bound weakly to γHV68 v-Bcl-2, with a K_d_ of greater than 300 μM. γHV68 v-Bcl-2 therefore differs significantly from Bcl-2 and Bcl-x_L_, which bind the Bad BH3 peptide with an affinity of 0.6–15 nM [9]. These results show that the γHV68-v-Bcl2 binds BH3 peptides from some, but not all, BH3 domain containing proapoptotic Bcl-2 family members.

### A Common Mechanism for Binding of BH3 Peptides by γHV68 v-Bcl-2 and Bcl-x_L_

**Figure 4:**
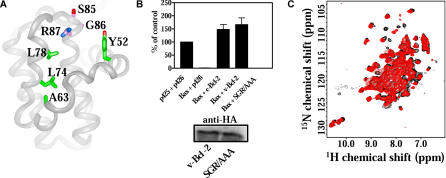
Mutagenesis of γHV68 v-Bcl-2 BH3 Binding Groove (A) Ribbon representation of γHV68 v-Bcl-2 showing residues that contact Bak peptide (in green and R87) and the SGR residues that were mutated (in magenta). (B) Growth of yeast following transformation with the indicated constructs (above, bar graph) and Western blot of SGR/AAA and wild-type v-Bcl-2 from transformed yeast (below, blot). (C) Overlay of the ^15^N-HSQC spectra of SGR/AAA (red contours) and wild-type (black contours) v-Bcl-2 showing conservation of structure between mutant and wild-type proteins.

The retention of a hydrophobic groove and the capacity to bind BH3 peptides by γHV68 v-Bcl-2 suggested that γHV68 v-Bcl-2 shares with Bcl-2 and Bcl-x_L_ a common capacity for binding BH3 peptides. However, the lack of Bad BH3 peptide binding led us to question whether the mechanism of BH3 peptide binding is shared between host Bcl-2 family proteins and the γHV68 v-Bcl-2. To directly address this question, we used nuclear Overhauser effect (NOE) experiments (Figure 4). We found that the Bak BH3 peptide bound to γHV68 v-Bcl-2 and Bcl-x_L_ in the same orientation ([53] and unpublished data), with contact occurring primarily between hydrophobic residues of v-Bcl-2 and Bak peptide. γHV68 v-Bcl-2 residues with NOE contacts to the Bak peptide (Figure 4A) were structurally homologous to those in the complex of Bcl-x_L_ and Bak peptide, in which Arg139 of Bcl-x_L_ forms a key contact with Asp83 of the Bak peptide [53].

Arg139 is highly conserved among Bcl-2 family proteins, and mutation of Asp83 in the Bak peptide to alanine (D83A) reduces its affinity for Bcl-x_L_ by more than 120-fold. The γHV68 v-Bcl-2 residue that is structurally homologous to Arg139 of Bcl-x_L_ is Arg87, which is a conserved amino acid in the v-Bcl-2 BH1 domain (see Figure 3D). We tested the importance of this Arg:Asp interaction in the binding of γHV68 v-Bcl-2 to Bak peptide in an NMR-based titration of v-Bcl-2 with D83A mutant Bak peptide. We observed no binding of D83A mutant Bak peptide to γHV68 v-Bcl-2 over the concentration range of the titration, indicating a K_d_ of more than 300 μM. Thus, Arg87 of γHV68 v-Bcl-2 played a key role, similar to that of Bcl-x_L_ Arg139, in binding to Asp83 of the Bak BH3 peptide. These data show that the molecular mechanisms of BH3 peptide binding are shared between γHV68 v-Bcl-2 and host antiapoptotic proteins.

### Residues in the γHV68 v-Bcl-2 BH3 Binding Groove Are Required for v-Bcl-2 Binding to BH3 Peptides and Inhibition of Bax Toxicity in Yeast

The conservation of the mechanism by which γHV68 and host Bcl-2 family members bind BH3 peptides suggested that the γHV68 v-Bcl-2 would also inhibit the function of proapoptotic Bcl-2 family members. We tested this hypothesis using a Bax toxicity assay in yeast (Figure 4). Inhibition of Bax toxicity in yeast by Bcl-2 family proteins depends on amino acids within the BH3 binding groove of the antiapoptotic family member and, at least in part, on heterodimerization with Bax [60–63].

The γHV68 v-Bcl-2 inhibited Bax-mediated death as effectively as Bcl-2 (Figure 4B). No inhibition of Bax-mediated death was observed in empty vector control transformations (unpublished data). Because structural data suggested the importance of the v-Bcl-2 Arg87, we tested the effect of mutating Arg87 and two adjacent residues (i.e., Ser85-Gly86-Arg87; in magenta in Figure 4A) to alanine (hereafter termed SGR/AAA) on the capacity of the v-Bcl-2 to inhibit Bax-mediated death. Mutations in this region have been shown to alter the antiapoptotic activity of both host and other viral Bcl-2 proteins [3,4,7,27]. We mutated all three amino acids based on preliminary studies demonstrating partial effects of single mutations in this region (unpublished data). The SGR/AAA mutation abrogated γHV68 v-Bcl-2 inhibition of Bax-mediated toxicity in yeast despite equivalent expression levels of wild-type and SGR/AAA mutant v-Bcl-2 protein (Figure 4B). Moreover, SGR/AAA mutant γHV68 v-Bcl-2 bound Bax peptide at least 1500-fold less well than wild-type γHV68 v-Bcl-2, as measured by NMR (K_d_ of greater than 300 μM compared to less than 5 μM for wild-type γHV68 v-Bcl-2). Analogous mutations in Bcl-2 and Bcl-x_L_ similarly abrogate antiapoptotic activity and binding to proapoptotic proteins [64,65]. Importantly, a comparison of the ^15^N- heteronuclear single-quantum coherence (HSQC) spectra of wild-type and SGR/AAA mutant γHV68 v-Bcl-2 confirmed that SGR/AAA mutant v-Bcl-2 retains the overall fold of wild-type v-Bcl-2 (Figure 4C), indicating that functional defects in SGR/AAA mutant γHV68 v-Bcl-2 are attributable specifically to alterations in the BH3 binding groove rather than to misfolding of the mutant protein. Thus, residues that lie in the γHV68 v-Bcl-2 BH3-binding groove were essential for both binding of Bax BH3 peptide and for inhibition of Bax-mediated toxicity in yeast.

### A Functional BH3 Binding Groove Is Essential for Efficient Reactivation of γHV68 from Latency and for Persistent γHV68 Replication

The conservation of the structure and function of the γHV68 v-Bcl-2 BH3 binding groove led us to hypothesize that this groove, and the conserved Arg87 required for interaction with BH3 peptides from proapoptotic Bcl-2 proteins and for inhibition of Bax toxicity in yeast, would be essential for the function of the v-Bcl-2 in vivo during γHV68 infection. To test this hypothesis, we compared the phenotypes of wild-type γHV68 virus, a γHV68 virus lacking the entire v-Bcl-2 due to a null mutation in the *M11* gene encoding v-Bcl-2 (Δv-Bcl-2) [36], and γHV68 viruses expressing the SGR/AAA mutant form of the v-Bcl-2.

**Figure 5:**
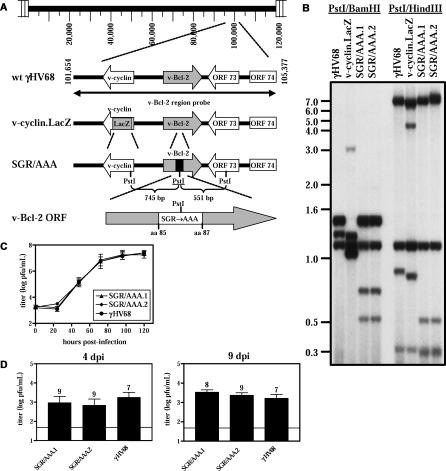
SGR/AAA Mutant Viruses Replicate Normally In Vitro and In Vivo (A) Shown are schematic illustrations of the genomes of γHV68, v-cyclin.LacZ, and SGR/AAA mutant viruses with the engineered PstI site underlined, and v-Bcl-2 containing the SGR/AAA mutation. (B) Southern blot of γHV68, v-cyclin.LacZ, and SGR/AAA mutant viruses. Expected bands (kb) for PstI/BamHI digest: γHV68, 1.5, 1.3, and 1.2; v-cyclin.LacZ, 1.3, 1.2, 1.1, and 0.05; and SGR/AAA, 1.5, 1.2, 0.8, and 0.6. Expected bands (kb) for PstI/HindIII digest: γHV68, 7.5, 1.2, 0.9, and 0.4; v-cyclin.LacZ, 7.1, 4.3, 1.2, 0.9, 0.4, and 0.07; and SGR/AAA, 7.5, 1.2, 0.6, 0.39, and 0.36. (C) Multistep growth curves of SGR/AAA mutants and γHV68. (D) Acute splenic titers at 4 or 9 dpi from mice infected with SGR/AAA mutants.

We generated two independent isolates of γHV68 containing the SGR/AAA mutation in the *M11* gene (SGR/AAA.1 and SGR/AAA.2; Figure 5A and 5B). SGR/AAA v-Bcl-2 mutant viruses displayed normal growth in fibroblast cells in vitro (Figure 5C), and replicated normally in the spleen (Figure 5D) and liver (unpublished data) of infected mice at 4 and 9 d post-infection (dpi). These results were expected, because the Δv-Bcl-2 γHV68 mutant virus, which lacks v-Bcl-2 expression, also exhibits completely normal replication during acute infection [36]. Together, these data indicate that the v-Bcl-2 does not have an essential function during γHV68 acute infection. The use of two independently generated mutants to analyze the phenotype attributable to the SGR/AAA mutation is an accepted standard in the field [36,66–68]. The equivalent phenotypes of SGR/AAA.1 and SGR/AAA.2 in multiple assays argues against the possibility that these phenotypes are due to chance mutations elsewhere in the viral genome.

**Figure 6:**
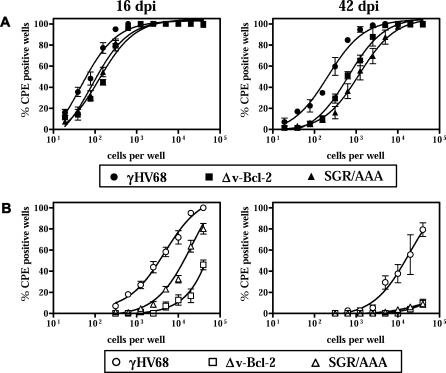
SGR/AAA Mutant Viruses Exhibit Defects in Chronic Infection In Vivo Ex vivo reactivation (A) and persistent replication (B) of γHV68, Δv-Bcl-2, or SGR/AAA mutant viruses at 16 or 42 dpi of IFNγ^−/−^ mice. No significant differences were observed between the two independent isolates of SGR/AAA mutants, and data from these two groups were pooled.

As the Δv-Bcl-2 mutant γHV68 virus has defects in reactivation from latency and in persistent replication [36], we tested the effects of the SGR/AAA v-Bcl-2 mutation on chronic γHV68 infection of IFNγ^−/−^ mice. IFNγ^−/−^ mice clear acute γHV68 infection normally [42,69], but exhibit increases in persistent replication and in the efficiency with which latently infected cells reactivate virus ex vivo [36,42]. Both Δv-Bcl-2 and SGR/AAA mutant γHV68 viruses exhibited small but statistically significant decreases in the frequency of cells that reactivate from viral latency ex vivo compared to wild-type γHV68 (Figure 6, Table 1). Furthermore, SGR/AAA mutant γHV68 viruses, like the Δv-Bcl-2 mutant γHV68 virus, showed no persistent replication at 42 dpi (Figure 6B, Table 1). The effect of the SGR/AAA v-Bcl-2 mutation on persistent γHV68 virus replication was significant, but less than the effect of a null mutation in the gene at 16 dpi. This partial phenotype was not due to reversion of the mutation in vivo, since 20 individual SGR/AAA mutant γHV68 viruses present in these mice were confirmed to have the mutation by nucleotide sequencing (unpublished data). Together, these data demonstrate that amino acids that are critical for the capacity of γHV68 v-Bcl-2 to bind BH3 peptides via the BH3 binding groove are essential for optimal reactivation from latency and for the capacity to persistently replicate in tissues at a low level after acute viral infection is contained by the immune response.

**Table 1:**
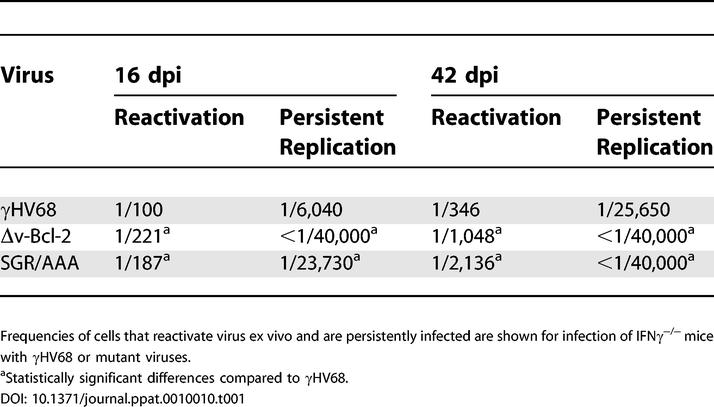
Summary of Chronic Infection Results

## Discussion

In this study, we combined structural and biochemical approaches to identify a BH3 peptide binding groove on the surface of the γHV68 v-Bcl-2 protein and amino acids within this groove that are essential for the capacity of the v-Bcl-2 to bind BH3 peptides. These same amino acids are essential for the capacity of the γHV68 v-Bcl-2 to block cell death in yeast induced by expression of the proapoptotic Bcl-2 family protein Bax, and for the contribution of the v-Bcl-2 to two fundamentally important aspects of γHV68 pathogenesis: reactivation from viral latency and persistent replication. Together, these data show that this domain of the v-Bcl-2 is essential for function in vivo, and support the concept that the γHV68 v-Bcl-2 functions in vivo by binding BH3 domains of antiapoptotic Bcl-2 family members.

### Presence of a Functional BH3 Binding Groove on γHV68 v-Bcl-2

We found that the γHV68 v-Bcl-2 shares with Bcl-2, Bcl-x_L_, and the KSHV v-Bcl-2 a BH3 binding groove [9]. Each of these proteins interacts with BH3 peptides from Bak and Bax, but the viral proteins differ from the host proteins in their inability to bind the Bad BH3 peptide [2,9]. Interestingly, the preference for Bak and Bax peptides over BH3 peptides from other proapoptotic family members is also seen for the adenovirus v-Bcl-2 [7].

These differences in BH3 peptide specificity between viral and host proteins have implications for how v-Bcl-2 proteins inhibit apoptosis. Some models of antiapoptotic Bcl-2 family protein function require that they heterodimerize with BH3-only proteins such as Bad in order to sequester these proapoptotic molecules away from Bak and Bax [3,4,57,59]. While we have not examined binding of BH3 peptides from BH3-only proteins other than Bad, the striking lack of binding of Bad BH3 peptide to γHV68 v-Bcl-2 suggests that the viral protein may not use this mechanism. Instead, these data support an alternative model that has also been proposed for cellular antiapoptotic Bcl-2 family proteins, but that is not generally accepted [3,4]. In this model, the antiapoptotic proteins directly target Bax and Bak rather than sequestering BH3-only proteins.

An interesting question is why KSHV and γHV68 v-Bcl-2 proteins bind the BH3 peptide from Bad with much lower affinity than is observed for Bcl-2 or Bcl-x_L_ [9]. In this property, the γHV68 and KSHV v-Bcl-2 proteins resemble cellular Mcl-1, which also fails to bind Bad BH3 peptide and is important for development of B cells, one of the cell types in which γHV68 establishes latency [70,71]. One possible explanation for the lack of binding to the Bad BH3 peptide is that v-Bcl-2 binding to Bad is detrimental to the virus. Bad was recently found to be essential for assembly of a holoenzyme that regulates mitochondrial respiration, and Bad deficiency results in defects in glycolysis and in aberrant glucose homeostasis [5]. Thus, v-Bcl-2 proteins may bind poorly to Bad, and perhaps other BH3-only Bcl-2 family proteins, to avoid interfering with functions of BH3 domain containing proteins in cellular processes other than induction of apoptosis. This would be important for a virus, such as γHV68, that expresses the v-Bcl-2 in latently infected cells [40], and would presumably benefit by not having v-Bcl-2 interfere with cellular processes essential for cell viability.

### γHV68 v-Bcl-2 Inhibits Apoptosis Induced by a Wide Array of Proapoptotic Stimuli

γ-herpesviruses maintain latent infection in lymphocytes for the life of the host, and must survive in the face of the immune response that effectively clears acute infection. These viruses then reactivate from latency and productively infect cells, yielding low levels of infectious virus in tissues, a process we refer to as persistent replication. It seems reasonable to speculate that latently infected cells may be exposed to multiple different proapoptotic stimuli over time. Furthermore, during the process of reactivation and persistent replication, infected cells are likely subjected to immune attack by lymphocytes that use proapoptotic cytokines or granule contents to kill infected cells. It is therefore not surprising that a protein such as the γHV68 v-Bcl-2, which is important during chronic infection, has the capacity to inhibit cell death induced by a wide variety of different stimuli, including antigen receptor crosslinking, corticosteroids, and γ-irradiation. In addition to the studies presented here, observations in cultured cells show that the γHV68 v-Bcl-2 inhibits apoptosis induced by TNFα, Fas, and Sindbis virus infection [37–39]. While it is possible that the γHV68 v-Bcl-2 utilizes different mechanisms to inhibit each of these death induction pathways, it is more reasonable to conclude that the viral protein inhibits a step in the death execution pathway common to all of these stimuli. In this regard, the capacity of the γHV68 v-Bcl-2 to bind BH3 peptides from Bax and Bak and to inhibit Bax-induced cell death in yeast is of particular interest. Bax and Bak are essential for induction of apoptosis through multiple pathways, and function therefore as a common gateway to the downstream steps in cell death execution [57–59]. Targeting these proteins would therefore have the advantage of protecting the infected cell from diverse proapoptotic insults.

Targeting Bax and Bak may be a common strategy for many viruses, since Bax and Bak are required for apoptosis during adenovirus infection, and the adenovirus v-Bcl-2 E1B 19K targets Bak and Bax for antiapoptotic function in cultured cells [7,72,73]. Furthermore, we believe that this is likely a general mechanism, since a role for BH1-domain amino acids in binding BH3 peptides has also been demonstrated for the adenovirus v-Bcl-2 and the African swine fever virus v-Bcl-2 family protein A179L [7]. Given the overall poor amino acid homology between viral Bcl-2 proteins and cellular Bcl-2 family members, the conservation of a mechanism for BH3 peptide binding between cellular and viral Bcl-2 family proteins strongly supports the hypothesis that this property of the v-Bcl-2 is important for function. This concept is supported by data provided here demonstrating the essential role of this domain in the function of the γHV68 v-Bcl-2 in vivo.

### Is the Critical In Vivo Role of Amino Acids in the BH3 Binding Groove Explained by v-Bcl-2 Binding to Proapoptotic Bcl-2 Family Proteins?

We found that amino acids in the BH3 binding groove of the γHV68 v-Bcl-2 are essential for in vivo function of the v-Bcl-2 protein. The major known function of the BH3 binding groove is interaction with BH3 domains of proapoptotic Bcl-2 family proteins, providing the basis for our conclusion that our data are consistent with a role for this biochemical function of v-Bcl-2 during chronic infection. However, there are other potential explanations for our results. It is possible that binding of BH3 domains is important for function, but that binding Bcl-2 family members is not. For example, non-Bcl-2 family proteins can contain BH3-like domains [16,17], and it is possible that the γHV68 Bcl-2 functions by binding to these proteins rather than to Bcl-2 family proteins.

Alternatively, the portion of the BH3 peptide binding groove that we mutated in the studies presented here might interact with other proteins that do not contain BH3 domains. Support for this possibility comes from the structure of the EBV v-Bcl-2 BHRF1, which is antiapoptotic but does not have an intact BH3 binding groove and does not measurably bind BH3 peptides [10]. It is possible that this v-Bcl-2, which lacks a BH3 binding groove, inhibits apoptosis by a mechanism distinct from that used by the γHV68 and KSHV v-Bcl-2 proteins, both of which have functional BH3 binding grooves. For example, the EBV v-Bcl-2 might inhibit apoptosis via interactions with other proteins such as Aven, Apaf-1, Btf, Beclin1, Raf-1, calcineurin, tissue transglutaminase, FAST, or p53 [6,16–26], all of which have been shown or suggested to to interact with host antiapoptotic Bcl-2 family members. Perhaps a capacity to interact with one or more of these proteins despite the SGR/AAA mutation explains the partial phenotype of the SGR/AAA mutant γHV68 viruses in persistent replication observed 16 dpi. It will be interesting to compare the antiapoptotic mechanisms of the γHV68 and KSHV v-Bcl-2 proteins with that of BHRF1 in order to determine whether interaction with proteins that do not contain BH3 is important for v-Bcl-2 function.

### Differences between Host and Viral Antiapoptotic Proteins

While γHV68 v-Bcl-2 shares an overall fold with its host counterparts, there are differences between the γHV68 v-Bcl-2 and host Bcl-2 family members, most notably the absence of a long loop between α1 and α2 [2]. Interestingly, the KSHV and EBV v-Bcl-2 proteins also lack this loop [9,10]. In cellular Bcl-2 family proteins, this loop contains sites for caspase cleavage and regulatory phosphorylation [1,2,54], and a portion of the loop has been predicted to be involved in p53 binding [19].

In contrast to host Bcl-2 family proteins [54,74], the KSHV and EBV v-Bcl-2 proteins are resistant to caspase cleavage [27,37], a process that generates proapoptotic products. Although γHV68 v-Bcl-2 also has a shortened α1/α2 loop, it does contain a caspase cleavage site (LDCV; see Figure 3D) and can be cleaved by caspases [37]. However, the predicted cleavage product does not have proapoptotic activity [37]. The absence of the α1/α2 loop in KSHV v-Bcl-2 also confers resistance to regulatory phosphorylation by the KSHV v-cyclin-CDK6 complex, which inactivates Bcl-2 [75,76]. Based on its structure, the γHV68 v-Bcl-2 is also expected to be refractory to regulatory phosphorylation. Thus, our structural analysis of the γHV68 v-Bcl-2 provides additional evidence that v-Bcl-2 proteins have evolved strategies to protect themselves from host cell regulatory mechanisms. The lack of an intact α1/α2 loop in v-Bcl-2 proteins decreases their susceptibility to inactivation via caspase cleavage or phosphorylation, an evolutionary strategy that we and others [2,6,7] speculate provides an advantage for the viruses by removing the antiapoptotic function of viral Bcl-2 family proteins from host cell control.

### Implications for Control of Chronic γ-Herpesvirus Infection

Most of the disease burden of human γ-herpesvirus infection occurs during chronic infection. Studies here demonstrating that amino acids in the BH3 binding groove of the v-Bcl-2 are essential for chronic viral infection raise the possibility that pharmacologic inhibition of v-Bcl-2 BH3 binding groove function might inhibit chronic infection. There may be sufficient differences between viral and host proteins' BH3 binding grooves to allow specific targeting of v-Bcl-2 proteins. Therefore, v-Bcl-2 proteins may provide a suitable therapeutic target for preventing or ameliorating γ-herpesvirus disease.

## Materials and Methods

### Animals.

Mice were housed and bred in a specific pathogen-free environment at Washington University School of Medicine in accordance with all federal and university policies. v-Bcl-2 transgenic mice were generated as previously described [77]. Briefly, the v-Bcl-2 ORF (genome coordinates 103418–103930 bp) was PCR-amplified and ligated into the BamHI site of the p1017 vector containing the hGH enhancer and the *lck* proximal promoter [78,79]. The construct was confirmed by DNA sequencing, and the DNA fragment used for microinjection was isolated by SpeI restriction digest followed by gel purification. B6 embryo manipulations were performed as previously described [77]. Transgene-positive F_1_ mice were identified by PCR and confirmed by Southern blot (unpublished data). Mice were maintained as heterozygotes on the B6 background and were genotyped by PCR using primers specific for the hGH enhancer within the transgene construct [77]. Nontransgenic littermates were used as negative controls for v-Bcl-2 transgenic mice. IFNγ^−/−^ mice on the B6 background were obtained from Jackson Laboratories (Bar Harbor, Maine, United States) and bred at Washington University. B6 mice were purchased from Jackson Laboratories. All studies were performed using age- and sex-matched mice between 8 and 12 wk of age.

### Quantitative RT-PCR.

Thymi and spleens from euthanized mice were disrupted over a 100 μm Nytex filter to generate single-cell suspensions. Total RNA was prepared from dissociated cells, and 2 μg was used in reverse transcription reactions to generate cDNA template for quantitative real-time PCR. Six replicate reactions of real-time PCR were performed per sample using a BioRad iCycler (BioRad, Hercules, California, United States) [80]. v-Bcl-2 specific primers were 5′-TAACATTGACCCAGGAGTTTAG-3′ and 5′-CGAGGTGAAAAGTTTGGAC-3′, and control reactions utilized primers specific for 18S RNA. No positive signal was detected in RNA samples to which reverse transcriptase was not added (unpublished data). Three transgene-positive mice from each founder line were analyzed, and nontransgenic samples consisted of one transgene-negative mouse from each founder line. v-Bcl-2 message copy number was calculated from a standard curve generated using 10^1^ to 10^5^ copies of a plasmid containing v-Bcl-2, and was normalized to 18S RNA levels.

### Flow cytometry.

Dissociated thymocytes were pre-incubated with anti-CD32/CD16 antibodies (Caltag Laboratories, Burlingame, California, United States; #MM7400) and stained with FITC-conjugated anti-CD4 (Pharmingen, San Diego, California, United States; #09424A) and tricolor-conjugated anti-CD8α (Caltag, #RM2206) antibodies. Cells were washed, fixed, and analyzed on a FACS Calibur. Data were analyzed using CellQuest (Becton Dickinson, Palo Alto, California, United States) and represent the mean ± SEM from at least two independent experiments. Statistical significance was determined using the Mann-Whitney test.

### Bax toxicity in yeast.

Yeast strain W303 was a generous gift from S. Zheng. Murine Bax and Bcl-2 clones were generous gifts from S. Korsmeyer. Bax was cloned into p425 GPD (ATCC, Manassas, Virginia, United States; #87359) and Bcl-2 was cloned into p426 GPD (ATCC #87361). SGR/AAA mutant v-Bcl-2 was generated using the ExSite PCR-Based Site-Directed Mutagenesis Kit (Stratagene, La Jolla, California, United States). Wild-type and SGR/AAA v-Bcl-2 were amino-terminal HA-tagged and cloned into p426 GPD. All constructs were verified by DNA sequencing, and 1 μg of each construct was used to transform yeast using the lithium acetate method. The number of colonies were counted 3 d following plating on selection media and is expressed as a percentage of that for a control transformation with p425 GPD and p426 GPD empty vectors. Transformations of yeast with each single construct resulted in growth only on the appropriate selection media (unpublished data). Data represent the mean ± SEM from four independent experiments, and statistical significance was determined using the Mann-Whitney test. For Western blots, total protein from transformed yeast was prepared using Y-PER Yeast Protein Extraction Reagent (Pierce Biotechnology, Rockford, Illinois, United States), resolved on an SDS-PAGE gel, and detected with an anti-HA antibody (Covance, Princeton, New Jersey, United States; #MMS-101P).

### Cell culture and viruses.

NIH 3T12 cells and MEFs were cultured in DMEM containing 10% fetal calf serum (D10). SGR/AAA mutant viruses were generated by homologous recombination as previously described [36]. Briefly, a targeting construct was generated by replacing wild-type v-Bcl-2 sequence with that for the SGR/AAA mutant in pL3700, which contains a 3,723-bp BamHI/BsrGI fragment of the γHV68 genome (genome coordinates 101,654–105,377 bp). The mutation generates a new PstI restriction site, which was used to distinguish wild-type from SGR/AAA viruses by Southern blot. The targeting construct was verified by DNA sequencing and cotransfected into 3T12 cells with viral DNA from v-cyclin.LacZ, which contains a LacZ expression cassette inserted into the adjacent v-cyclin ORF [81]. Recombinant “white” viruses were isolated following X-Gal staining and screened by Southern blot for the SGR/AAA mutation in v-Bcl-2. Two independent isolates were generated from separate cotransfections, and each isolate was subjected to three rounds of plaque purification. Fifteen individual plaques from each isolate were genotyped by Southern blot to confirm the absence of contamination with either wild-type or parental v-cyclin.LacZ viruses (unpublished data), and one of the tested plaques was used to generate virus stocks of each isolate. γHV68 clone WUMS (ATCC VR1465), Δv-Bcl-2 [36], and SGR/AAA mutants were subcultured and titered by plaque assay on 3T12 cells as previously described [82]. For in vitro multistep growth curves, 3T12 cells were infected at a MOI of 0.05. Samples were harvested at various time points postinfection, subjected to three freeze-thaw cycles, mechanically disrupted with 1 mm silica beads, and titered by plaque assay [36].

### In vivo infection.

Mice were intraperitoneally injected with 10^6^ PFU of virus in 0.5 ml of D10. For acute titers, half a spleen and half a lobe of liver per mouse were mechanically disrupted with 1 mm silica beads and titered by plaque assay [81]. Data represent the mean ± SEM from at least two independent experiments, and statistical significance was determined using the Mann-Whitney test. For ex vivo reactivation, peritoneal cells pooled from 3–5 mice per experimental group were plated in serial 2-fold dilutions onto MEF monolayers, which were assessed for cytopathic effect caused by reactivated virus after 21 d. Persistent replication was analyzed by quantitating preformed infectious virus in mechanically disrupted cells, which cannot reactivate virus [83]. Data represent the mean ± SEM from at least three independent experiments, and were analyzed by nonlinear regression using GraphPad Prism (GraphPad Software, San Diego, California, United States). The frequency of cells that reactivated virus ex vivo was determined by calculating the cell density at which 63.2% of wells were positive for cytopathic effect. Statistical significance was determined using a paired t-test.

### v-Bcl-2 expression and purification.

Wild-type and SGR/AAA v-Bcl-2 used in NMR studies were expressed in *E. coli* BL21(DE3) strain grown on M9 media. For wild-type v-Bcl-2, uniformly ^15^N-labeled, uniformly ^15^N,^13^C-labeled, and uniformly ^15^N,^13^C-labeled, 75% ^2^H samples were prepared with media containing either ^15^NH_4_Cl, ^15^NH_4_Cl plus [U-^13^C]glucose or ^15^NH_4_Cl, [U-^13^C]glucose, and 75% ^2^H_2_O, respectively. For SGR/AAA v-Bcl-2, a ^15^N-labeled sample was prepared with media containing ^15^NH_4_Cl as the sole nitrogen source. Soluble protein was purified by Ni^2+^-affinity chromatography. NMR samples contained 0.5–1.0 mM protein in either 90% H_2_O with 10% ^2^H_2_O or 100% ^2^H_2_O, 20 mM ^2^H-TRIS (pH 7.8), and 5 mM ^2^H-dithiothreitol.

### NMR spectroscopy.

All NMR experiments were acquired at 303 K on a Bruker DRX500, DRX600 or DRX800 NMR spectrometer. Backbone ^1^H, ^13^C, and ^15^N resonance assignments were achieved with (^15^N,^13^C,[75%]^2^H) γHV68 v-Bcl-2 using a suite of deuterium-decoupled, triple-resonance experiments (HNCA, HN[CO]CA, HN[CA]CB, HN[COCA]CB, HNCO and HN[CA]C) [84,85]. Side-chain ^1^H and ^13^C NMR signals were assigned from HCCH-TOCSY experiments [86]. Stereospecific assignments of valine and leucine methyl groups were obtained from an analysis of the ^13^C-^13^C coupling patterns observed for biosynthetically directed, fractionally ^13^C-labeled γHV68 v-Bcl-2 [87]. NOE distance restraints were obtained from three-dimensional ^15^N- and ^13^C-edited NOESY spectra [88,89] acquired with a mixing time of 80 ms. Slowly exchanging amide protons were identified in an ^15^N-HSQC spectrum recorded immediately after exchanging the protein into a buffer prepared with ^2^H_2_O.

### Structure calculations.

v-Bcl-2 structures were calculated using a simulated annealing protocol [90] with the program CNX (MSI, San Diego, California, United States). A square-well potential (F_NOE_ = 50 kcal mol^−1^) was employed to constrain NOE-derived distances. Based on cross-peak intensities, NOE-derived distance restraints were given upper bounds of 3.5, 4.5, or 6.0 H. In the refinement stage, additional ambiguous constraints were added, with an upper bound of 6.0 H, for unassigned cross peaks that were consistent with the chemical shift table (i.e., error bars of 0.07 ppm for protons and 0.7 ppm for hetero atoms) and the structure. Torsion angle restraints, φ and ψ, were generated from analysis of N, C′, C^α^, and H^α^ chemical shifts using the TALOS program [91]. A force constant of 200 kcal mol^−1^rad^−2^ was applied to all torsional restraints. Explicit hydrogen bonds were included in α-helices only for residues observed to have slowly exchanging amide protons. The program PROCHECK was employed to analyze the geometric quality of the calculated structures in the ensemble [92].

### Peptide binding.

Binding of the Bak 16-mer (GQVGRQLAIIGDDINR), the Bax 16-mer (KKLSECLKRIGDELDS), the Bad 25-mer (NLWAAQRYGRELRRMSDEFVSFKK), and a D83A mutant Bak 16-mer (GQVGRQLAIIGADINR) to wild-type v-Bcl-2, and binding of the Bax 16-mer to SGR/AAA v-Bcl-2 was assessed by NMR titration [93]. Each peptide was titrated from a concentrated stock solution into a sample of ^13^C-labeled protein, and binding was monitored from changes in a ^13^C methyl-HSQC spectrum [93]. For wild-type v-Bcl-2, ^13^C-HSQC spectra were recorded on a uniformly ^13^C,^15^N-labeled protein sample (120 μM) in the presence of increasing amounts of peptide (40, 80, 160, 240, and 320 μM). For SGR/AAA v-Bcl-2, ^15^N-HSQC spectra were recorded on a sample of uniformly ^15^N-labeled protein in the presence of increasing amounts of peptide.

## Supporting Information

Figure S1 v-Bcl-2 Inhibits Cell Death in Thymocytes Representative dot plots showing CD4 and CD8 profiles of nontransgenic or v-Bcl-2 transgenic thymocytes after no treatment or treatment with 0.3 mg of dexamethasone, 250 rads of γ-irradiation, or 30 μg of anti-CD3ɛ antibody.
(174 KB PDF)

Figure S2 Solution Structure of γHV68 v-Bcl-2 Ribbon representations of (A) γHV68 v-Bcl-2 and (B) Bcl-x_L_. Helices are numbered with respect to Bcl-x_L_. The BH1, BH2, BH3, and BH4 regions are colored magenta, red, green and yellow, respectively. Connolly surface for (C) γHV68 v-Bcl-2 and (D) Bcl-x_L_. The Connolly surface was calculated using a probe radius of 1.4 Å. Residues are colored as follows: Leu, Val, Ile, Phe, Tyr, Trp, Met, and Ala are yellow; Arg, Lys, and His are blue; Asp and Glu are red; and all other residues are gray. Black arrows indicate the hydrophobic grooves present on the surfaces of Bcl-x_L_ and γHV68 v-Bcl-2.
(5.3 MB PDF)

## Abbreviations

BH: Bcl-2 homology
dpi: days post-infection
EBV: Epstein-Barr virus
γHV68: murine γ-herpesvirus 68
HSQC: heteronuclear single-quantum coherence
IFNγ: interferon-γ
KSHV: Kaposi's sarcoma-associated herpesvirus
NMR: nuclear magnetic resonance
NOE: nuclear Overhauser effect
v-Bcl-2: viral Bcl-2 homolog
