# Supplementary figures and images for: A Surface Groove Essential for Viral Bcl-2 Function During Chronic Infection In Vivo
Source: PLoS Pathog. 2005 Sep 30;1(1):e10. doi: 10.1371/journal.ppat.0010010 (PMC1238745; doi:10.1371/journal.ppat.0010010)

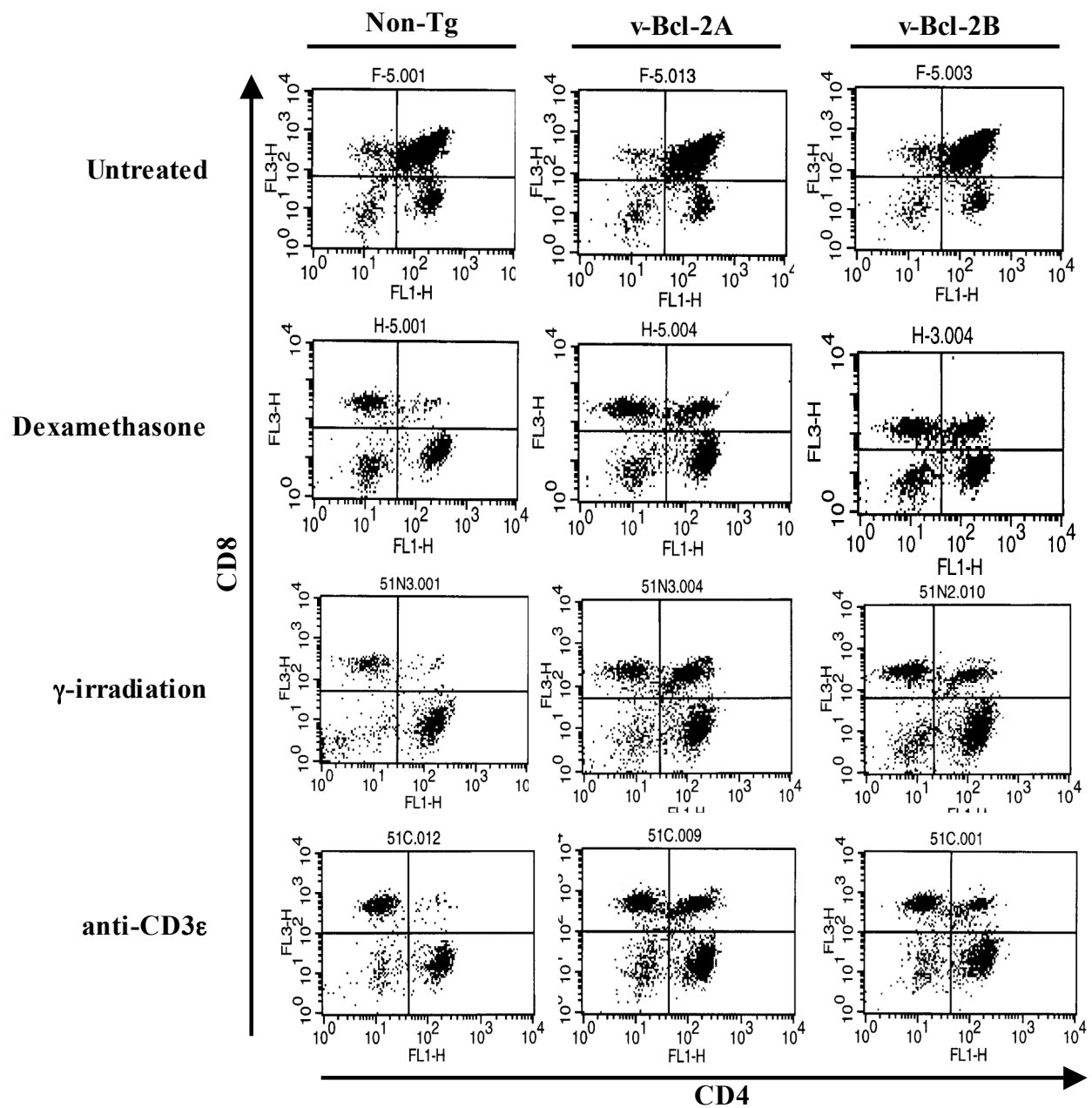

Supplement: Figure S1 — Representative dot plots showing CD4 and CD8 profiles of nontransgenic or v-Bcl-2 transgenic thymocytes after no treatment or treatment with 0.3 mg of dexamethasone, 250 rads of γ-irradiation, or 30 μg of anti-CD3ɛ antibody. (174 KB PDF) [file ppat.0010010.sg001.pdf]

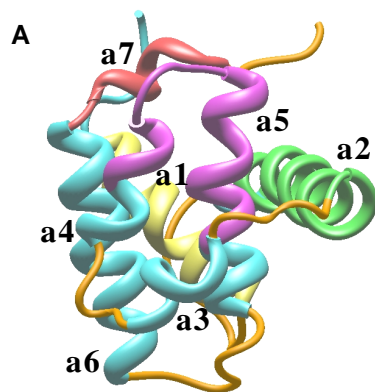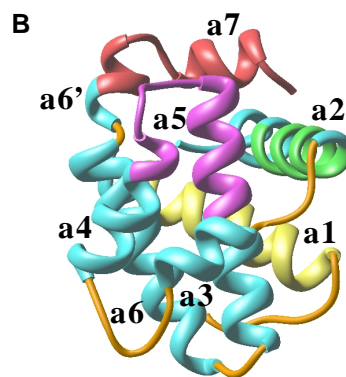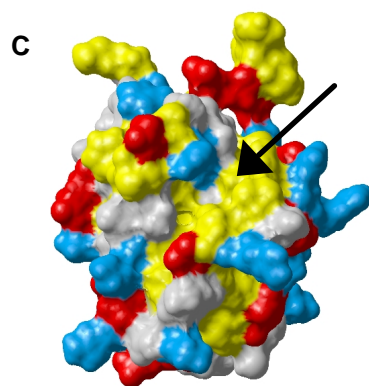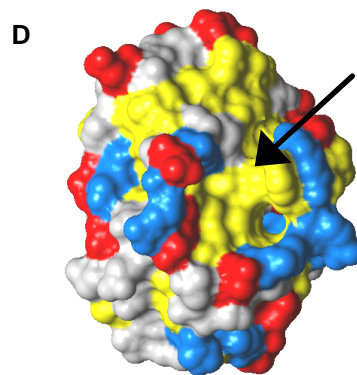

Supplement: Figure S2 — Ribbon representations of (A) γHV68 v-Bcl-2 and (B) Bcl-xL. Helices are numbered with respect to Bcl-xL. The BH1, BH2, BH3, and BH4 regions are colored magenta, red, green and yellow, respectively. Connolly surface for (C) γHV68 v-Bcl-2 and (D) Bcl-xL. The Connolly surface was calculated using a probe radius of 1.4 Å. Residues are colored as follows: Leu, Val, Ile, Phe, Tyr, Trp, Met, and Ala are yellow; Arg, Lys, and His are blue; Asp and Glu are red; and all other residues are gray. Black arrows indicate the hydrophobic grooves present on the surfaces of Bcl-xL and γHV68 v-Bcl-2. (5.3 MB PDF) [file ppat.0010010.sg002.pdf]
